# WIN55,212-2-Induced Expression of Mir-29b1 Favours the Suppression of Osteosarcoma Cell Migration in a SPARC-Independent Manner

**DOI:** 10.3390/ijms20205235

**Published:** 2019-10-22

**Authors:** Antonietta Notaro, Sonia Emanuele, Fabiana Geraci, Antonella D’Anneo, Marianna Lauricella, Giuseppe Calvaruso, Michela Giuliano

**Affiliations:** 1 Department of Biomedicine, Neurosciences and Advanced Diagnostics (BIND), University of Palermo, 90127 Palermo, Italy; antonietta.notaro@unipa.it (A.N.); sonia.emanuele@unipa.it (S.E.); marianna.lauricella@unipa.it (M.L.); 2 Department of Biological, Chemical and Pharmaceutical Sciences and Technologies (STEBICEF), University of Palermo, 90128 Palermo, Italy; fabiana.geraci@unipa.it (F.G.); antonella.danneo@unipa.it (A.D.); giuseppe.calvaruso@unipa.it (G.C.)

**Keywords:** osteosarcoma, cannabinoids, cell migration, miR-29b1, SPARC

## Abstract

WIN55,212-2 (WIN) is a synthetic agonist of cannabinoid receptors that displays promising antitumour properties. The aim of this study is to demonstrate that WIN is able to block the migratory ability of osteosarcoma cells and characterize the mechanisms involved. Using wound healing assay and zymography, we showed that WIN affects cell migration and reduces the activity of the metalloproteases MMP2 and MMP9. This effect seemed to be independent of secreted protein acidic and rich in cysteine (SPARC), a matricellular protein involved in tissue remodeling and extracellular matrix deposition. SPARC release was indeed prevented by WIN, and SPARC silencing by RNA interference did not influence the effect of the cannabinoid on cell migration. WIN also increased the release of extracellular vesicles and dramatically upregulated miR-29b1, a key miRNA that modulates cell proliferation and migration. Interestingly, reduced cell migration was observed in stably miR-29b1-transfected cells, similarly to WIN-treated cells. Finally, we show the absence of SPARC in the extracellular vesicles released by osteosarcoma cells and no changes in SPARC level in miR-29b1 overexpressing cells. Overall, these findings suggest that WIN markedly affects cell migration, dependently on miR-29b1 and independently of SPARC, and can thus be considered as a potential innovative therapeutic agent in the treatment of osteosarcoma.

## 1. Introduction

Osteosarcoma (OS) is a primary malignant bone tumour that most often affects males with two incidence peaks, one of which is between 10 and 40 years of age and the other in the elderly [1,2]. In particular, the marked osteoblastic and osteogenic activity of adolescent age seems to be a predisposing factor for the onset of the neoplasia [3]. The first indication of the use of chemotherapy in patients with OS dates back to 30 years ago, and there is currently a full consensus in considering the combination of surgery and chemotherapy as a standard procedure in treating high-grade OS. However, although the tumour can respond to chemotherapy treatment, in patients with metastatic disease, the prognosis remains adverse [4]. Thus, a better understanding of osteosarcoma biology represents an important challenge for researchers to optimize treatment strategies and develop new therapeutic agents, thus improving the prognosis.

Cannabinoids (CBs), the active constituents of *Cannabis sativa*, are known to exert a wide range of neuronal central and peripheral effects. Recently, a role of cannabinoids in the regulation of cell death and survival has largely emerged [5,6,7]. In particular, numerous studies have explored the anti-proliferative effects of these compounds in various tumours [8]. Following the interaction with their specific receptors, cannabinoids can trigger several different signalling pathways [9], including the accumulation of ceramide, the activation of c-Jun N-terminal kinase (JNK) and p38 MAPK, as well as the increase in calcium concentration, reactive oxygen species (ROS) production and the modulation of pro- and anti-apoptotic members of the Bcl-2 family [10,11,12,13]. More recently, a relationship between the cannabinoid system and miRNA expression has been evidenced. MiR-Let-7d has been demonstrated to be a target of cannabinoid receptors [14], and the anticancer activity of WIN was related to the miR-27a-mediated repression of specificity protein (Sp) transcription factor in colon cancer cells [15].

In recent years, oncology research has investigated specific aspects of the cellular processes involved in cell death caused by synthetic cannabinoid derivatives with anticancer activity. Our previous studies have shown the in vitro effects of the synthetic cannabinoid WIN55,212-2 on different cancer cell lines [16,17,18]. In particular, we showed that treatment with increasing doses of WIN induces a significant reduction of the proliferative ability of MG63 osteosarcoma cells and sensitizes them to apoptosis induced by TRAIL, a cytokine with selective anticancer activity [19]. The analysis of biochemical pathways evidenced an important role played by the WIN-dependent increase in the control of the intracellular level of SPARC (secreted protein acidic and rich in cysteine), a multi-faceted glycoprotein which is involved in a number of cellular processes [20,21].

In addition to pro-apoptotic and anti-proliferative roles, other studies reported that synthetic cannabinoids can also reduce the migration, angiogenesis, invasion and metastasis of cancer cells by modulating the levels of proteins involved in these processes [22]. On the other hand, it is well known that SPARC participates in the regulation of cell adhesion, migration, and tissue remodelling [23]. In particular, it has been shown that low expression levels of matricellular SPARC can modulate cell migration in different types of cancer cells, and these observations led researchers to hypothesize a specific role of SPARC in the inhibition of tumour progression and invasiveness. In contrast, many other studies demonstrated an oncogenic function of SPARC, thereby highlighting the divergent roles of SPARC in human carcinogenesis [24].

Other interesting players in the regulation of cell migration in different types of cancer cells are the members of the miR-29 family, including products from two gene loci: miR-29a/b1, located on chromosome 7 (7q 32.2); and miR-29b2/c, located on chromosome 1 (1q 32.2) [25]. It has been demonstrated that the forced overexpression of miR-29 is able to inhibit cell migration and proliferation and promote the apoptosis of tumour cells, while its reduced levels are a frequent occurrence in osteosarcoma tissues [26,27]. However, although it is known that miR-29 is a regulator of SPARC expression [28], the exact correlation between these two players in cannabinoid action is a subject of investigation.

The aim of the present study was to evaluate the effects of the cannabinoid WIN in osteosarcoma MG63 cell migration and the possible involvement of SPARC and miR-29b1 in this event. Collectively, our results show for the first time that WIN is able to inhibit osteosarcoma cell migration in a SPARC-independent manner. Moreover, a crucial role seems to be played by the WIN-mediated induction of miR-29b1. Therefore, the cannabinoid has the potential to be an efficient anti-cancer drug in new therapeutic strategies for osteosarcoma.

## 2. Results

### 2.1. WIN Treatment Reduces the Migratory Ability of MG63 Cells and Affects MMP Activity

**Figure 1:**
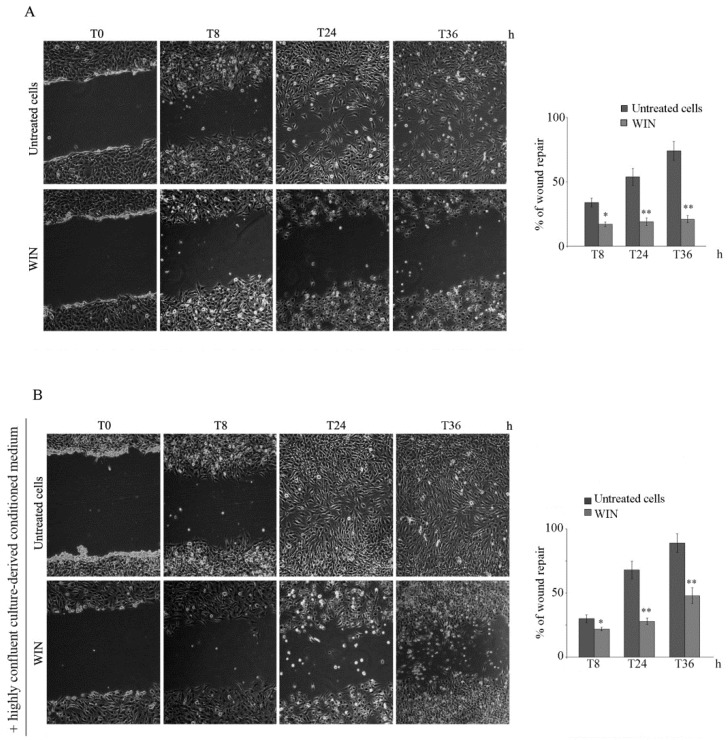
WIN55,212-2 (WIN) treatment inhibits MG63 cell migration. The effect of WIN on MG63 wound healing in the absence (**A**) or presence of conditioned medium (**B**). After mechanical wounding, confluent cells were treated with 5 µM WIN and cell migration was monitored over time. In (**B**), before treatment, cell culture medium was replaced with conditioned medium from high confluence cultures. Representative microphotographs were taken at the indicated time points after wounding (magnification 200×). The average distances between cells at the edges of the gaps in the wound healing assay were estimated by ImageJ software. Histograms reporting the mean percentage area healed at T8, T24 and T36 post-wounding are shown on the right panels. (*) *p* < 0.05 and (**) *p* < 0.01 versus untreated cells at the same time point.

In a previous paper, we demonstrated the ability of WIN—a synthetic agonist of cannabinoid receptors—to induce a significant reduction of osteosarcoma cell proliferation, and we characterized the biochemical mechanisms involved [19]. Here, we aim to evaluate whether WIN can also induce a modification in the migratory ability of osteosarcoma MG63 cells. To this end, a monolayer of cells was scraped longitudinally, and the rate of the re-population of the area between the wound edges after the lesion was evaluated. Untreated MG63 cells showed a rapid healing process of the lesion, reaching almost full monolayer repair at 48  h (T48) after scratching (not shown). As shown in Figure 1A, 5 µM WIN markedly prevented cell migration, as indicated by the entity of the wound that was almost identical to T0 at all time points observed. In contrast, untreated MG63 cells migrated in the gap, reaching about 75% of migratory activity after 36 h (T36).

In other experiments, before WIN treatment, we added a conditioned medium from a highly confluent untreated cell culture to the cultured cells. As shown in Figure 1B, the presence of conditioned medium accelerated the wound closure of untreated cell cultures, almost achieving total confluence at T36, and partially prevented the anti-migratory effect induced by WIN. These data suggest that the cells produce factors to sustain cell migration and that WIN treatment blocks their extracellular release or reduces their action.

**Figure 2:**
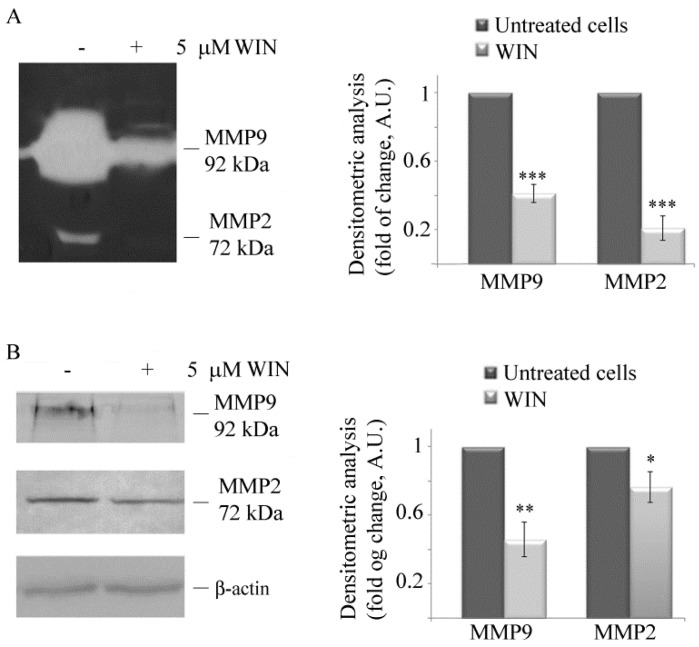
WIN treatment inhibits metalloprotease activities. Gelatin zymography (**A**) and Western blotting analysis (**B**) of the metalloproteinases MMP2 and MMP9 in MG63 cells treated for 36 h with 5 µM WIN. Arrows indicate the relative bands at 72 kDa (MMP2) and 92 kDa (MMP9). Blots are representative of three independent experiments with similar results. Densitometric analysis is reported in the histograms. (*) *p* < 0.05, (**) *p* < 0.01 and (***) *p* < 0.001 compared to the untreated sample. In (**A**), volumes containing equal amount of proteins were loaded, in (**B**), densitometric measurements were made after normalization with β-actin.

Next, we analysed the levels and the activity of metalloproteinases (MMPs), a family of enzymes which are responsible for normal tissue remodelling and angiogenesis. Gelatin zymography assay showed that 5 µM WIN significantly reduced extracellular MMP9 and MMP2 gelatinolytic activity after 36 h of treatment, thus indicating that WIN inhibits metalloproteinase activity (Figure 2A). In addition, Western blot analysis revealed that WIN was capable of inducing a dramatic decrease in the level of intracellular MMP9 and a slight decrease in the level of MMP2 (Figure 2B).

### 2.2. WIN Treatment Prevents SPARC Release in the Extracellular Environment and Increases the Release of Extracellular Vesicles

**Figure 3:**
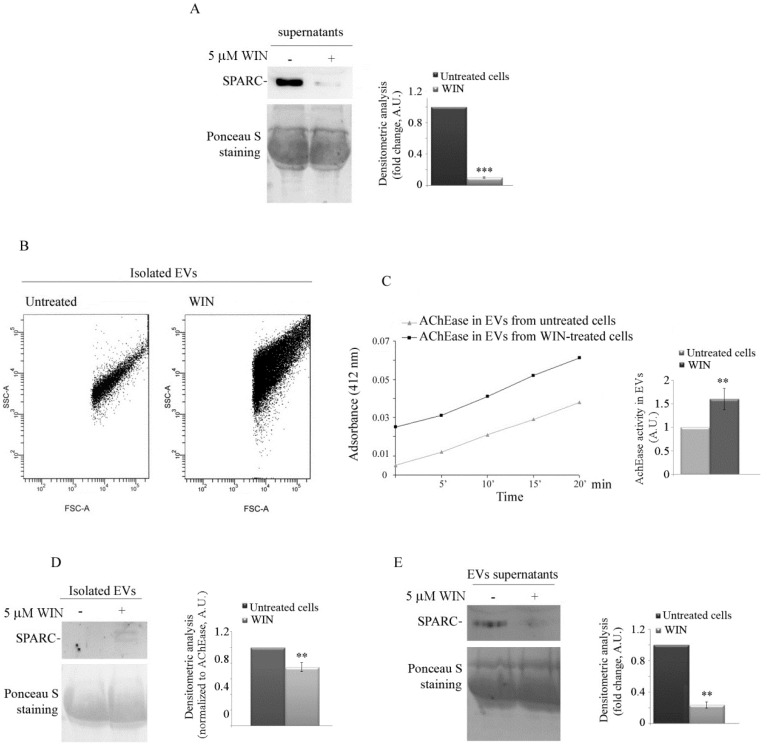
WIN treatment blocks secreted protein acidic and rich in cysteine (SPARC) release and increases the number of extracellular vesicles. (**A**) Western blotting analysis of SPARC in media derived from untreated or WIN-treated cells for 36 h. The media were dialyzed, concentrated by lyophilization, and reconstituted with water. Protein samples were separated under reducing conditions, and immunoblotting was performed using SPARC antibody. Red Ponceau staining was reported as loading control. The results are representative of three independent experiments and densitometric analysis is reported in the histograms. (***) *p* < 0.001 compared to untreated cells. (**B**) Flow cytometry analysis of isolated extracellular vesicles (EVs) derived from untreated or WIN-treated MG63 cells. Data are representative of three independent experiments. SSC, side scatter; FSC, forward scatter. (**C**) Acetylcholine esterase (AChEase) activity in EVs from untreated or WIN-treated cells assessed as reported in Section 4 and relative quantification. (**D**,**E**) Western blotting analysis of SPARC in MG63 cell-derived EVs. Media from untreated or WIN-treated cells were collected and EVs isolated as reported in Section 4. The immunoblotting of isolated EVs (**D**) or EV supernatants (**E**) was performed to detect SPARC level. AchE activity (**D**) and Ponceau red staining (**E**) were used as loading control. Representative blots of three independent experiments and densitometry analysis histograms are depicted. (**) *p* < 0.01 compared to the untreated sample.

Among the numerous players implicated in regulating tumour cell migration and invasiveness, we specifically focused on SPARC protein and miR-29b1. The matricellular factor SPARC plays different roles in extracellular processes, and its function is strictly related to the cancer model and/or the metastatic grade of the tumour [29]. Since we previously demonstrated that, in osteosarcoma cells, the cytotoxic effect of WIN was accompanied by an increase in the level of SPARC [20], here, we aim to analyse a possible role of this factor in the anti-migratory effect of WIN in MG63 cells. To this end, we first analysed the level of SPARC in conditioned media collected from untreated or WIN-treated cells after dialysis and lyophilization. Although we have previously demonstrated that intracellular levels of SPARC increase under treatment with WIN [20], Western blotting analysis evidenced that the level of released SPARC was much more abundant in the medium from untreated cells compared to that obtained from WIN-treated cells (Figure 3A).

In light of this observation, we wondered whether SPARC release was associated with extracellular vesicles (EVs). Therefore, we isolated EVs from the culture supernatants of untreated or WIN-treated MG63 cells by ultracentrifugation and quantified EVs obtained by both cytofluorimetry and acetylcholine esterase activity measurement (AChEase). As shown in Figure 3B, WIN induced a considerable increase in the amount of released EVs, which resulted to be about seven-fold higher than EVs isolated from untreated cell media. This result was confirmed by the consistent increase in AChEase activity (Figure 3C).

Subsequently, we evaluated SPARC levels in EVs by Western blotting analysis. The results showed a not significant level of SPARC in the vesicles, although the protein was present in the relative supernatants from untreated cell cultures (Figure 3D,E).

**Figure 4:**
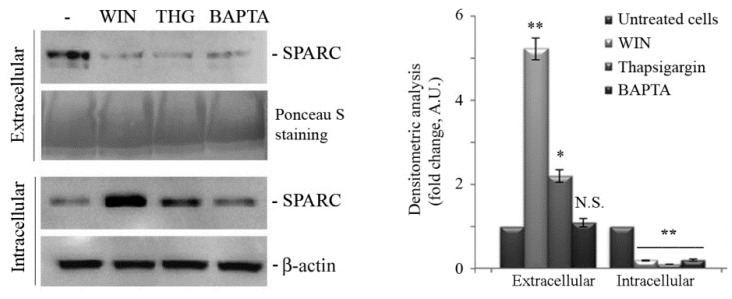
Effects of secretory pathway inhibitors on SPARC levels. Western blotting analysis of extracellular or intracellular level of SPARC after treatment with 5 µM WIN, 1 µM thapsigargin (THG) or 5 µM BAPTA-AM (BAPTA). Ponceau red staining or β-actin blot were reported as loading control for extracellular or intracellular protein levels, respectively. Representative blots of three independent experiments and densitometry analysis histograms are depicted. (*) *p* < 0.05, (**) *p* < 0.01 compared to the untreated sample. N.S. not significant.

Therefore, considering that SPARC is poorly represented in EVs, we evaluated whether this protein can be secreted by MG63 cells via the canonic secretory pathway. For this purpose, we used thapsigargin and BAPTA-AM, two specific inhibitors of this pathway. As shown in Figure 4, the level of SPARC was significantly reduced in the medium from thapsigargin or BAPTA-AM-treated cells. Accordingly, in the presence of secretory pathway inhibitors, the intracellular levels of SPARC were higher than in control cells (Figure 4). Thus, we concluded that WIN could restrain SPARC inside the cell by inhibiting the secretory pathway and that the effect of WIN on cell migration is independent of the presence of SPARC in the extracellular compartment.

### 2.3. Silencing SPARC did not Affect the Anti-Migratory Effect of WIN

**Figure 5:**
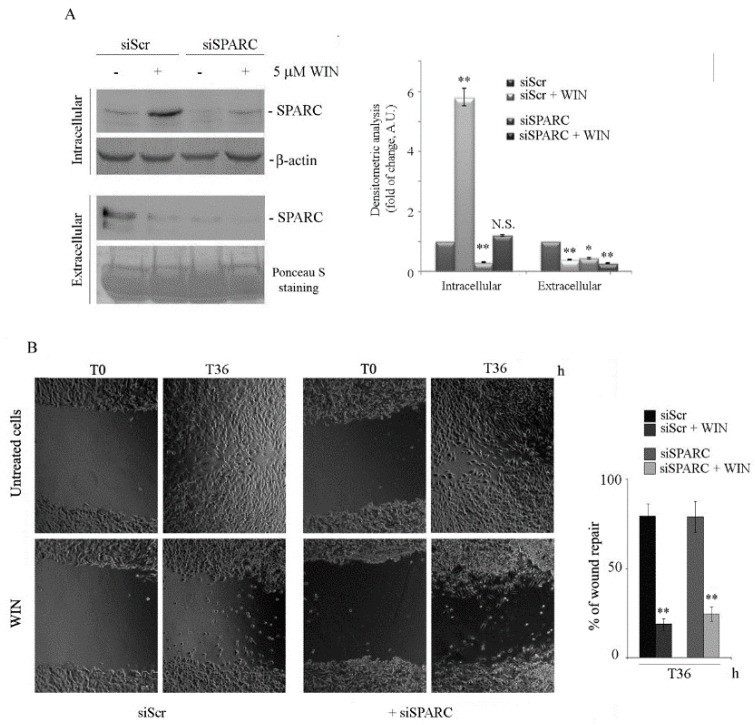
The effect of WIN on MG63 cell migration is not dependent on SPARC. (**A**) The effect of SPARC siRNA (siSPARC) on the protein level under 5 µM WIN treatment in both intracellular or extracellular fractions. β-actin blot or Ponceau red staining were reported as a loading control for intracellular or extracellular protein levels, respectively. Representative blots of three independent experiments and densitometry analysis histograms are depicted. (*) *p* < 0.05, (**) *p* < 0.01 compared to the untreated sample at the same time point. N.S. not significant. (**B**) Effects of siSPARC on cell migration in comparison with scramble control (siScr). Pictures reported were taken at T36 after wounding (magnification 200×). The average distances between cells at the edges of the gaps in the wound healing assay were estimated through ImageJ software. On the right panel, a graph of the mean percentage area healed at T36 post-wounding is shown. (**) *p* < 0.01 compared to untreated cells at the same time point. N.S. not significant.

To validate the hypothesis that SPARC is not involved in the WIN-mediated anti-migratory effect, we silenced SPARC expression by RNA interference. Figure 5A shows that SPARC was downregulated in silenced cells at both the intracellular and extracellular level in the presence or absence of WIN. The analysis of cell migration in SPARC-silenced cells was then evaluated. The results reported in Figure 5B show that—also in this case—WIN almost completely prevented the closure of the wound after 36 h in both control (siScr-transfected) and siSPARC-transfected cells. In the absence of the cannabinoid, transfected cells closed the gap with a migratory activity of about 80% regardless of the level of SPARC.

**Figure 6:**
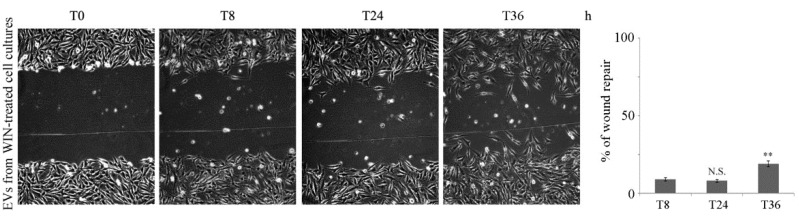
Extracellular vesicles from WIN-treated cultures halt MG63 cell migration. Wound healing migration assay of MG63 cells in the presence of EVs (50 µg/mL) isolated from WIN-treated cells. The average distances between cells at the edges of the gaps in the wound healing assay were estimated through ImageJ software. Pictures reported were taken at different time points after wounding (magnification 200×). On the right of the picture a graph of the mean percentage area healed at T8, T24 and T36 post-wounding is shown. (**) *p* < 0.01 compared to the original gap at T0. N.S. not significant.

Considering the substantial increase in the amount of released EVs after WIN treatment, we evaluated whether EVs play a role in the control of cell migration. For this purpose, MG63 cells were incubated in the presence of the EVs isolated from conditioned medium of WIN-treated cell cultures. As shown in Figure 6, EVs isolated from WIN-treated cells halted the wound closure similarly to that observed after WIN treatment, whereas EVs isolated from untreated cultures did not induce any modification in cell migration (not shown).

### 2.4. WIN Induces the Upregulation of miR-29b1 which Plays a Role in Cell Migration

**Figure 7:**
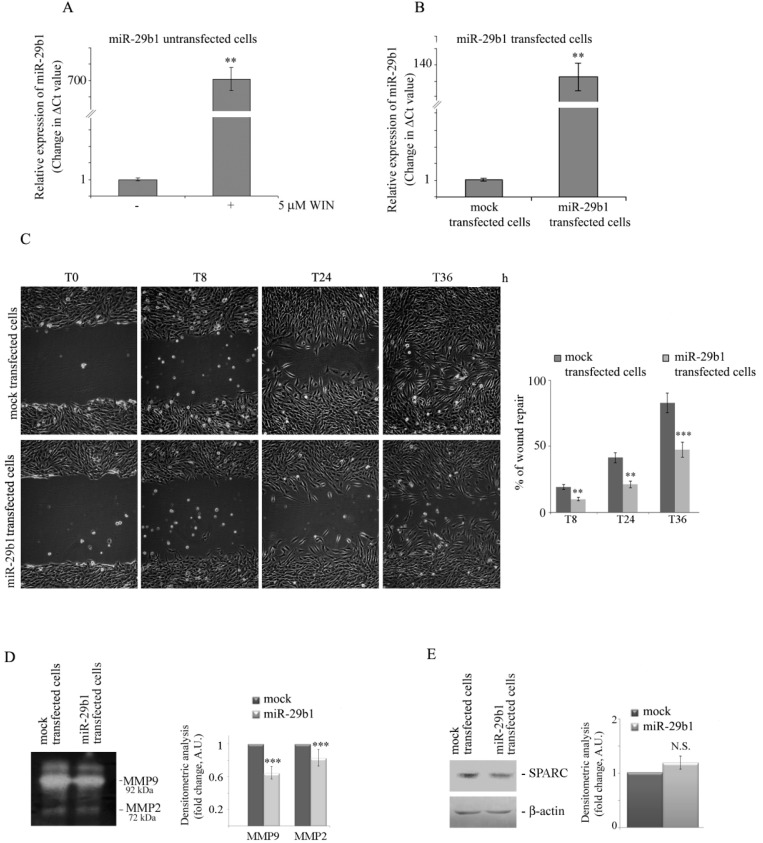
WIN induces a dramatic increase in miR-29b1 expression, and miR-29b1 overexpression reduces cell migration. (**A**) Gene expression levels of miR-29b1 in untreated or 5 µM WIN-treated cells for 24 h were analysed by RT-qPCR. (**) *p* < 0.01 compared to untreated cells. (**B**) Validation of gene expression changes in cells stably transfected with miR-29b1 versus mock transfected cells. The height of each column reflects the ratio of the expression of miR-29b1 compared to U6 (a non-coding small RNA used as a control) (**) *p* < 0.01 and (***) *p* < 0.001 compared to the untreated cells at the same time point. (**C**) Wound healing migration assay of miR-29b1-transfected MG63 cells in comparison with cells transfected with mock alone. Pictures were taken at the indicated time points after wounding (magnification 200×). The average distances between cells at the edges of the gaps in the wound healing assay was estimated through ImageJ software. On the right of the picture, a graph of the mean percentage area healed at T8, T24 and T36 post-wounding is shown. (**) *p* < 0.01 and (***) *p* < 0.001 compared to the untreated cells at the same time point. (**D**) Gelatin zymography of MMP2 and MMP9 in miR-29b1-transfected cells versus mock transfected cells. Arrows indicate the relative bands at 72 kDa (MMP2) and 92 kDa (MMP9). Volumes containing equal amount of proteins were loaded. Densitometry values are averaged from three independent experiments. (***) *p* < 0.001. (**E**) Western blotting analysis of SPARC level in miR-29b1 overexpressing cells. The results were obtained by immunoblotting employing a specific SPARC antibody as reported in Section 4. β-actin blot was included as a loading control. Densitometry values are averaged from four independent experiments normalized to β-actin. N.S. not significant.

To explore the mechanism by which WIN prevents cell migration, we focused on miR-29b1, which is considered a mediator of the migratory ability of cancer cells [28,30]. Real-time PCR experiments showed that WIN induces an upregulation of this miRNA of about 700-fold compared with untreated MG63 cells (Figure 7A). To investigate the role of this miRNA on cell migration, we transfected MG63 cells with a vector carrying miR-29b1 and selected a stable line overexpressing this miRNA (Figure 7B). Then, we analysed the motility of these cells by means of wound healing assay. Similar to WIN-treated cells, after 36 h, only a small percentage of transfected cells migrated into the wound, whereas mock transfected cells showed a migratory activity of about 80% with respect to that observed in untreated cells (Figure 7C). Moreover, miR-29b1 transfected cells showed a reduced extracellular activity of matrix metalloprotease 9 and 2 (MMP9 and MMP2) (Figure 7D), mimicking the WIN effect. In addition, also the intracellular levels of both MMPs were reduced in miRNA transfected cells (not shown).

Since miR-29b1 is considered a putative post-transcriptional regulator of SPARC expression [30], we evaluated the level of SPARC in miR-29b1 overexpressing cells. As shown in Figure 7E, the overexpression of miR-29b1 did not cause any change in the level of SPARC, so we concluded that, in our experimental model, SPARC is not a direct target of miR-29b1, as predicted by in silico analysis.

## 3. Discussion

Cell migration represents an essential step during tumour progression, and therefore inhibitors of this process can be considered as potential antimetastatic drugs. On the basis of our previous results on apoptosis induction by WIN55,212-2 in osteosarcoma cells [19,20], in the current study, we show the ability of this compound to block osteosarcoma cell migration and we characterize the mechanism involved. Specifically, we demonstrate here that WIN markedly reduced the migratory ability of osteosarcoma MG63 cells, and this effect was accompanied by a dramatic reduction in the extracellular activity and intracellular levels of MMP2 and MMP9 metalloproteases. Although conflicting data are present in the literature regarding the expression levels of metalloproteases in osteosarcoma cells [31,32], we found that, in our serum-free conditions, both the intracellular levels and extracellular activity of MMP9 were higher than those of MMP2. Fragmentary data in the literature demonstrate that WIN can inhibit the epithelial mesenchymal transition and migration in different cancer cell models [33,34] In an attempt to clarify the mechanism of the anti-migratory effect of WIN in osteosarcoma cells, our study specifically focuses on a possible involvement of the matricellular protein SPARC and a hypothetical role exerted by miR-29b1. SPARC is often considered as an ambiguous gene product because it plays opposite roles in extracellular matrix remodelling and carcinogenesis [24]. Despite the fact that we have previously demonstrated a crucial role exerted by intracellular SPARC in WIN-induced apoptosis in osteosarcoma cells, in this paper, we demonstrate that SPARC is not involved in the anti-migratory effect of WIN. Indeed, although WIN upregulated intracellular SPARC, it markedly prevented its release and restrained the protein inside the cells by inhibiting the canonical secretory pathway. This conclusion is based on the observation that, in our model, WIN mimicked the effects of BAPTA-AM and thapsigargin, two selective inhibitors of the secretory pathway. We also demonstrate that WIN increased the production of extracellular vesicles, in which SPARC was not present. Moreover, as a confirmation that the anti-migratory effect of WIN was independent of SPARC, the knockdown of this factor by RNA interference did not influence the migratory ability of osteosarcoma cells either in the presence or absence of WIN.

In our opinion, an interesting point in this study is the evidence that WIN induced a marked increase in the amount of secreted EVs and that EVs isolated from WIN-treated cells exerted a significant anti-migratory effect in untreated cultures. Ongoing studies in our laboratory attempt to characterize by proteomic analysis the content of WIN-induced EVs and this will shed light on the factors involved in WIN-mediated intercellular communication.

**Figure 8:**
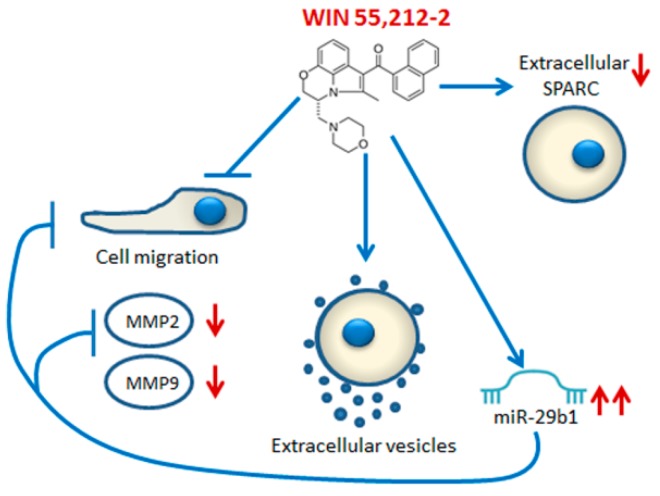
Schematic representation of the WIN-induced anti-migratory effect.

Osteosarcoma cells are characterized by the downregulation of miR-29 family members which exert an anti-proliferative and pro-apoptotic role in this cancer and sensitize the cells to the effects of chemotherapeutic agents [35]. Interestingly, we demonstrate that WIN induced a dramatic increase in the level of miR-29b1, and that the stable overexpression of this miRNA produced a decrease in cell migration and a reduction of MMP2 and MMP9 activity similar to those observed in WIN-treated cells. This represents the first evidence that miR-29b1 is able to induce the downregulation of both MMP2 and MMP9. Figure 8 shows a schematic representation of the WIN-induced mechanism responsible for its anti-migratory effect in osteosarcoma MG63 cells. The preventive role of miR-29b1 on cell migration is in accordance with data recently reported by Zhu et al. [36] which demonstrate a role of miR-29 in the regulation of osteosarcoma cell proliferation and migration in connection with CDK6. Our current study aims to verify the presence of miR-29b1 in WIN-induced EVs and discriminate the vesicle types. Moreover, another intriguing aspect concerns the relationship between cannabinoid effects and the specific receptors (CB1-R and/or CB2-R) expressed in the different cancer models [37]. In this regard, we consider it relevant to characterize the cannabinoid receptor in osteosarcoma cells that is responsible for the anti-migratory effect of WIN.

In conclusion, considering that the five-year survival rate in metastatic osteosarcoma is about 15–30% and that no specific drug has been found to date, the understanding of molecular mechanisms that regulate osteosarcoma migration and the potential role exerted by the cannabinoid WIN may have important implications for more specific osteosarcoma treatment.

## 4. Materials and Methods

### 4.1. Reagents

R-[2,3-Dihydro-5-methyl-3[(4-morpholinyl)methyl]pyrrolo[1,2,3,-de]-1,4-benzoxazin-6-yl]-1-naphthalenyl methanone mesylate (WIN55,212-2) was purchased from Sigma, (Sigma Aldrich, Milan, Italy). Stock solutions were prepared in DMSO and opportunely diluted in culture medium. The final concentration of DMSO never exceeded 0.04%, which is a concentration that was experimentally determined to have no discernible effect. All the experiments were carried out using vehicle alone as a control. Antibody against SPARC was purchased from Takara (Takara Bio Clontech, Mountain View, CA, USA), anti β-actin from Sigma (Sigma Aldrich, Italy) and anti-MMP2 and MMP9 from Santa Cruz Biotechnology (Santa. Cruz, CA, USA).

### 4.2. Cell Cultures

Human osteosarcoma MG63 cells were acquired from Interlab Cell Line Collection (ICLC; Genoa, Italy). Cells were cultured at 37 °C in Dulbecco’s modified Eagle’s medium (DMEM), supplemented with 10% (*v*/*v*) heat-inactivated foetal bovine serum (FBS), 2.0 mM *L*-glutamine, and antibiotic antimycotic solution (100 U/mL penicillin, 100 µg/mL streptomycin and 250 ng/mL amphotericin B; Sigma, Milan, Italy) in a humidified atmosphere containing 5% CO_2_. For the experiments, cells were seeded in 96 or six-well plates, and after 24 h, culture medium was replaced with fresh serum-free DMEM before cannabinoid treatment.

### 4.3. Wound Healing Assay

Changes of migration and motility of cancer cells were examined using a wound-healing assay [38]. Cells (10^6^) were seeded in six-well dishes to achieve approximately 90% confluence. Using a sterile 200 µL pipette tip, a straight scratch simulating a wound in a monolayer was made. After scratching, wells were gently washed with medium to remove the detached cells, and fresh serum-free DMEM medium was added. MG63 cells were treated with the vehicle (untreated cells), 5 µM WIN or 50 µg/mL EVs. In other experiments, before WIN treatment, the medium was replaced with serum-free medium from highly confluent untreated cultures. The plates were then incubated at 37 °C, and the speed of cell movement across the gap was observed. Digital documentation at the same position was made after scratching at time zero (T0) and after 8, 24 and 36 h and captured by a computer-imaging system (Leica DC300F camera and Adobe Photoshop for image analysis). The effects on cell migration and motility were estimated by using ImageJ software (National Institutes of Health, Bethesda, MD, USA). The area of the remaining wound was determined as the ratio between the residual area at a given time point and the original wound area (T0) × 100.

### 4.4. Gelatin Zymography

The enzymatic activity of MMP2 and MMP9 was measured by gelatin zymography in 10% SDS-polyacrylamide separating gels in the presence of gelatin. Cells were treated with 5 µM WIN for 24 h, and the conditioned media from untreated or WIN-treated cells were collected and centrifuged (2000× *g* for 5 min) to remove cells and cell debris. The amount of total protein in the supernatants was assessed by the Bradford assay (Bio-Rad, Segrate, Milan, Italy) according to the manufacturer’s protocol. Supernatants were incubated with an adequate volume of SDS sample loading buffer without β-mercaptoethanol and separated by electrophoresis. Following electrophoresis, gels were rinsed with enzyme renaturing buffer containing 2.5% Triton X-100 in 50 mM Tris-HCl (pH 7.5) for 60 min at room temperature. Subsequently, gels were incubated in developing buffer (0.15 M NaCl, 10 mM CaCl_2_, in 50 mM Tris-HCl, pH 7.5) for 24 h at 37 °C, stained with Coomassie brilliant blue R-250 solution for 2 h and destained in methanol, acetic acid and water (50:10:40) solution until clear bands of MMPs activity were visible on the dark blue background. Digestive activity of MMPs was confirmed by the presence of two bands on zymograms, slower migrating MMP9 and faster migrating MMP2 in comparison with a standard of molecular weight.

### 4.5. Western Blot Analysis

To evaluate the extracellular protein content, conditioned culture media were dialyzed using 5000 cut off dialysis tubing and concentrated by lyophilization. Samples reconstituted with water were separated by electrophoresis under reducing conditions. For the evaluation of intracellular proteins, extracts were prepared as previously reported [39,40]. Ponceau red staining (for extracellular proteins), AChEase activity (for SPARC in EVs) or β-actin blots (for intracellular proteins) were reported as loading control. The blots were developed using the enhanced chemiluminescence (ECL) labeling system or alkaline phosphatase colorimetric system. Optical densities of the bands were analysed with Quantity One Imaging software from Bio-Rad Laboratories. The results shown in the figures are representative of at least three independent experiments with similar results.

### 4.6. Isolation and Quantification of Extracellular Vesicles from Culture Media

Extracellular vesicles (EVs) were purified from medium as previously described [41]. Briefly, conditioned media by subconfluent cells were centrifuged at 2000× *g* for 10 min and at 4000× *g* for 15 min to remove cells and large debris. The supernatant was ultracentrifuged in a 70-Ti rotor (Beckman Coulter, Brea, CA, USA), at 105,000× *g* for 90 min at 4 °C, and pelleted vesicles were resuspended in filtered PBS. The protein content of isolated EVs was determined using the Bradford method (Bio-Rad, Segrate, Milan, Italy) with bovine serum albumin as standard.

The number of obtained EVs was determined by flow cytometry with a FACSCanto instrument (BD Biosciences, Erembodegem, Belgium) as previously described. Briefly, 1 μL of EVs was diluted in a fixed volume of 200 μL of filtered PBS (0.1 μM filter); all samples were analysed by FACS for 30 s at medium flow rate. The event number corresponds to the number of EVs present in a specific volume of sample [42].

EVs release was also quantified by measuring the activity of acetylcholinesterase (AchEase) by Ellman assay [43]. Briefly, EVs (5 µL) were suspended in filtered PBS (95 µL) and incubated with 1.25 mM acetylthiocholine chloride (Sigma, Milan, Italy) and 0.1 mM 5,5′-dithio-bis(2-nitrobenzoic acid) (Sigma, Milan, Italy). PBS was then added to a final volume of 1 mL, and the change in absorbance at 412 nm was monitored every 5 min for 20 min.

### 4.7. Gene Silencing Using siRNA

Small interfering RNAs (siRNAs) against SPARC (5’-AACAAGACCUUCGACUCUUCC-3’) (siSPARC) and scrambled siRNA (siScr), as a negative non-silencing control, were purchased from Dharmacon RNA Technologies (Chicago, IL, USA). For the experiments, cells (10^5^) were seeded in six-well plates and cultured in antibiotic-free DMEM supplemented with 2.0 mM *L*-glutamine, until 50% confluence. Then, cells were transfected with 30 nM siSPARC or siScr in the presence of 5 µL Metafectene Pro (Biontex Laboratories GmbH, Martinsried/Planegg, Germany) in a final volume of 1 mL serum-free medium. The reaction was stopped after 6 h replacing the culture medium with fresh 10% FBS DMEM. After 48 h from transfection, silenced cells were treated with WIN for other 24 h.

### 4.8. Real-Time PCR for miR-29b1 Expression

RNA was extracted by Direct Zol RNA Mini-Prep (Zymo research, Freiburg, Germany). A DNase I treatment step was included. Twenty nanograms of total RNA was reverse transcripted in a final volume of 10 μL by using miRCURY LNA^TM^ Universal RT microRNA PCR kit (Exiqon, Mi, Italy) according to the manufacturer’s instructions. The resulting cDNAs were used for quantitative analysis by real-time PCR (qPCR) using miR-29b1 LNA™ primers (204261; Exiqon) and SYBR Green Master Mix (Exiqon). Reactions were performed in 96-well plates according to manufacturer’s instructions, using Bio-Rad instrument, as previously reported [44]. qPCR was performed in triplicate and repeated for confirmation. U6 small nuclear RNA was used as internal control for miRNA detection. Data processing and statistical analysis were performed by using IQ5 cycler software. The relative quantification in gene expression was determined using the 2^−ΔΔ*C*t^ method.

### 4.9. Stable Transfection of MG63 Cells with miR-29b1 Plasmid Vector

For the stable transfection of MG63 cells expressing miR-29b1, a specific plasmid construct, prepared as reported in [44], was employed. Before stable transfection, cells were seeded in six-well plates until they reached 90% confluence and then transfected with 4 µg of miR-29b1 expression vector or empty vector (mock) encoding the green fluorescent protein (GFP), by using Lipofectamine 2000 (Invitrogen^TM^, Monza, Italy) according to manufacturer’s instructions. The efficiency of transfection was evaluated by monitoring the ratio of transfected cells showing GFP fluorescence in relation to all cells. Two days after transfections, the cells were transferred in 100 mm dishes in selective medium containing 1 μg/mL Puromycin. The medium was replaced every 3–4 days. A plate of non-transfected cells was used as a control for the selection.

### 4.10. Statistical Analysis

Data, reported as means ± SD from at least three independent experiments, were analysed using the Student’s *t*-test. Differences were considered significant at *p* < 0.05.

## Abbreviations

SPARC	Secreted protein acidic and rich in cysteine
MMPs	Matrix metalloproteinases
EVs	Extracellular vesicles
AchEase	Acetylcholinesterase
THG	Thapsigargin
